# A multi-center trial of LGE-MRI of the left atrium

**DOI:** 10.1186/1532-429X-15-S1-O111

**Published:** 2013-01-30

**Authors:** Eugene Kholmovski, Kavitha Damal, Nathan S Burgon, Sathya Vijayakumar, Chankevin Tek, Anya G Mihaylova, Nassir F Marrouche

**Affiliations:** 1 CARMA Center, University of Utah, Salt Lake City, UT, USA; 2 UCAIR, Department of Radiology, University of Utah, Salt Lake City, UT, USA

## Background

Atrial fibrillation (AF) is the most common rhythm disturbance. Radio-frequency ablation (RFA) of the left atrium (LA) is effective for drug refractory AF patients. Outcome of RFA procedure depends on the degree of pre-ablation LA fibrosis and amount of post-ablation scar. Late gadolinium enhancement (LGE) imaging can detect fibrosis and visualize scar. However, only few centers with advanced expertise in cardiac MR (CMR) have demonstrated successful LGE of LA. Multi-center study was initiated to study reproducibility of LGE of LA.

## Methods

11 clinical centers with different degrees of CMR expertise and typical MRI hardware have participated in this study. Customized pulse sequences and imaging protocols for LGE of LA were installed on 13 Siemens scanners at the centers: 1.5T - 4 Avanto, 2 Espree, 1 Sonata, 1 Symphony; 3T - 3 Verio, 2 Trio. 6 centers used 1.5T scanners, 4 centers used 3T scanners and 1 center used both 1.5 and 3T. 3 centers used specialized cardiac coils; the others used standard body and spine coils. The participating centers followed their regular protocol for CMR contrast injection (Table 1). MRI technologists in most centers underwent 1-2 days training in LA imaging.

**Table 1:** Type of contrast agent and dosage in the participating sites

Contrast agent	The number of centers	Dose (mmol/kg)
Dotarem	1	0.2

Gadovist	3	0.15, 0.15, 0.2

Magnevist	4*	0.1, 0.2, 0.2, 0.2

Multihance	3*	0.1

Omniscan	1	0.2

*- one center switched from Magnevist to Mutihance during the study.

370 AF patients underwent LGE-MRI within 30 days prior to RFA. LGE-MRI was repeated for 270 AF patients at least 3 months after RFA to quantify post-ablation scar. Quality of LGE images was scored by two experienced blinded readers: good - 2, fair - 1, poor - 0. In cases of score discrepancy, consensus was achieved by the readers. Poor quality images were not clinically useable. Good and fair quality images can be used for fibrosis and scar quantification. Poor quality images and supporting data (heart rhythm and rate, navigator signal, etc.) were analyzed to find the main reason for scan failure.

## Results

The results of image analysis are summarized in Table 2. Quality was better for pre-ablation scans performed on 3T scanners than on 1.5T (p<0.01). Post-ablation scans consistently have better quality than pre-ablation scans (p<0.02). 13.0% of pre and 9.3% of post-ablation scans was graded as poor. In 62% of the cases MRI technologist error was the main reason for poor quality. Typical errors were wrong inversion time, wrong phase-encoding direction, partial coverage of LA, navigator prescription error, poor ECG signal, and error in the main frequency adjustment (only at 3T). In 31% cases, poor quality was patient related: significant arrhythmia, very irregular respiration, heart rate > 120 bpm, patient motion, and extreme obesity. Artifacts in parallel imaging reconstruction on Espree were responsible for poor quality in 7% cases.

**Table 2:** Image quality of pre- and post-ablation LGE-MRI of the left atrium

	Pre-ablation			Post-ablation		
Scanner	1.5T	3T	1.5 & 3T	1.5T	3T	1.5 & 3T

# scans	161	209	370	101	169	270

Good	39.8%	53.1%	47.3%	53.5%	59.8%	57.4%

Fair	43.5%	36.8%	39.7%	31.7%	34.3%	33.3%

Poor	16.7%	10.1%	13.0%	14.8%	5.9%	9.3%

Score	1.230±0.718	1.431±0.669	1.343±0.697	1.386±0.734	1.539±0.608	1.482±0.661

## Conclusions

The multi-center trial demonstrates that it is possible to consistently acquire clinically useable LGE of LA using customized sequences and imaging protocols in centers without advanced CMR expertise utilizing typical MRI hardware and contrast agents. Better training in LA imaging for MRI technologists, may further improve image quality of LGE of LA.

